# Hampstead General Hospital

**Published:** 1905-12-23

**Authors:** 


### HAMPSTEAD GENERAL HOSPITAL.

### OPENING OF THE NEW BUILDINGS.

H.R.H. Princess Christian visited Hampstead on Saturday afternoon, December 16, for the purpose of opening the new hospital on Haverstock Hill, the foundation-stone of which she had laid some two years ago. A very large assemblage of subscribers and sympathisers was gathered together in a marquee erected for the purpose in the grounds of the hospital. At 3.15 Princess Christian, having finished her tour of inspection of the hospital, entered the marquee, and took her seat on the platform. Mr. Ernest Collins, chairman of the Hospital Council, said that the pleasant office of welcoming their patron had fallen to him that day. The sympathy and support the Princess had given them on several previous occasions had been of the greatest encouragement to them in their efforts in this good cause. Having referred to the appreciation Princess Christian had shown during her visit to the wards, Mr. Collins went on to say that the hospital, as it stood, fully equipped and furnished, had cost £32,000, towards which they had obtained £26,000, and had therefore a debt of £6,000 still hanging over them. In addition to this there was a loan for the site due to one of their supporters of £4,000, so that the total amount of debt must be considered £10,000. It was not the intention of the governors to continue the extension of the hospital until the whole of this debt was cleared off. The extension would consist of wards providing beds for fifty free in-patients, and they also intended to build a wing for a paying ward. As arranged by the founder, Dr. Heath Strange, there would be a Nursing Home as well as a public hospital for the benefit of suffering humanity. The great support they had received in the past from the inhabitants of Hampstead, the generosity of both their President and treasurer, the munificent gifts from King Edward's Hospital Fund, and the large donation from the trustees of the Zunz bequest, all encuraged them in the belief that they would not have to wait in vain for the support which would relieve them of their debt, and thus set their energies free to continue the good work and complete the scheme as proposed.

Sir George Barham, the Mayor of Hampstead, remarked that the Princess was no stranger to Hampstead, and bade Her Royal Highness welcome to it in the name of the citizens.

### A Handsome Gift.

Sir Henry Harbin, President of the hospital, said that Princess Christian ought not to be styled the patron of the hospital merely, but its patron saint. Sir Henry then announced that if the Council would undertake to raise £5,000 within a short time he would promise on behalf of an anonymous donor the sum of £20,000 for the completion of the hospital. This announcement was greeted with loud applause by the large audience, and Sir Henry further explained that the £20,000 could be devoted to everything needed in connection with the hospital, such as furniture, fittings, etc. One great advantage in this gift would be this. Up to now they had not been able to do anything for the League of Mercy, but now that the ladies of Hampstead would not be obliged to work for the hospital, he begged them to proceed at once to get up a branch of the League of Mercy, and, knowing what he did of their energy and devotion, he was sure that in a short time they would have as good a branch as anywhere in the kingdom.

After a hymn had been sung, the Bishop of London read some prayers and pronounced a benediction upon the hospital.

Princess Christian then said: "I have great pleasure in declaring this hospital now open, and wish it all prosperity and all success."

Mr. J. S. Fletcher, M.P., wished Her Royal Highness a happy Christmas when it came, and recalled the many visits the Princess had paid Hampstead for philanthropic objects.

Her Royal Highness next unveiled a portrait of Dr. W. Heath Strange, the founder of the hospital, by Mr. Savage Cooper, and the proceedings terminated with the National Anthem.

A visit to the new hospital showed it to be equipped with all modern appliances. The bright, airy wards had already a good many patients in them, the total number of beds the present building accommodates being 64.

